# Comparative evaluation of sealing ability and adaptation of gel form of MTA to dentinal walls: an in-vitro study

**DOI:** 10.1038/s41405-024-00271-y

**Published:** 2024-11-24

**Authors:** Haritha Parthasarathy, Akshara Modak, Karuna Yarmunja Mahabala, Srikant Natarajan, Shrikala Baliga, Manuel Thomas, Ashwin Rao, Anupama Nayak

**Affiliations:** 1 Manipal College of Dental Sciences, Mangalore, Manipal Academy of Higher Education, Manipal, Karnataka 576104 India; 2 Department of Pediatric and Preventive Dentistry, Manipal College of Dental Sciences, Mangalore, Manipal Academy of Higher Education, Manipal, Karnataka 576104 India; 3 Department of Oral Pathology, Manipal College of Dental Sciences, Mangalore, Manipal Academy of Higher Education, Manipal, Karnataka 576104 India; 4 Department of Microbiology, Kasturba Medical College, Mangalore, Manipal Academy of Higher Education, Manipal, 576104 India; 5 Department of Conservative Dentistry and Endodontics, Manipal College of Dental Sciences, Mangalore, Manipal Academy of Higher Education, Manipal, Karnataka 576104 India

**Keywords:** Mineral trioxide aggregate, Root canal treatment

## Abstract

**Context:**

Mineral Trioxide Aggregate (MTA) is a calcium silicate-based cement that potentially exhibits improved washout resistance when carboxymethyl chitosan or gelatin is incorporated. Gel-form MTA is a novel mineral trioxide aggregate formulated using construction industry-based technology. The present study was conducted to comparatively evaluate the sealing ability and adaptation to dentinal walls of gel-form MTA.

**Materials and methods:**

This in-vitro study consisted of two groups: gel-form MTA and the conventional powder-liquid MTA. 10 samples per group were used for each of the tested parameters. Adaptation of the MTA to the dentinal walls was tested under the light microscope and measured using Image J software. Sealing ability was evaluated using a single aerobic bacterial leakage model. Appropriate statistical analysis was done for the obtained data. Adaptation of the MTA was analyzed using independent t-test and Friedman test, whereas the bacterial leakage was analyzed using chi-square test.

**Results:**

On comparison of the adaptation property at coronal and apical thirds, there was no statistically significant difference between the groups (*p* = 0.071 and *p* = 0.638, respectively). However, while comparing the same in the middle one-third of the root, lesser gaps were identified in the gel-form MTA group (*p* = 0.013). One sample belonging to the conventional powder-liquid MTA group showed significant turbidity during bacteria leakage evaluation (*p* = 0.001) with the presence of *E. faecalis* in the count of 10^3^ colony forming units/milliliter.

**Conclusion:**

The gel-form MTA shows a better adaptation to the dentinal walls at the middle third of the root and exhibits better sealing ability against bacterial leakage when tested for *E. faecalis*. The adaptation of gel-form MTA at coronal and apical third of the root was comparable to the conventional powder-liquid MTA.

## Introduction

Mineral Trioxide Aggregate (MTA) cements are mainly made up of tricalcium silicates and are radiopaque in nature. Upon addition of water, they form a self-setting calcium silicate hydrate gel [1]. The first production of MTA was patented in 1995, and it had bismuth oxide powder added to gray Portland cement as a radio pacifier [2]. Consequently, Primus proposed the white version of MTA, which was made of white Portland cement, without iron in its composition in 2004. MTA has certain favorable properties which make this cement suitable for use in a clinical environment [2, 3]. These properties include sealing ability, which can be directly correlated to the expansion; release of hydroxyl and calcium ions which creates an alkaline environment, thereby, preventing the proliferation of disadvantageous bacteria; the ability to set in the presence of fluids; and the capacity to form a mineralized barrier into the surrounding environment [4].

Initially, due to the wet sand-like consistency after manipulation with water, clinicians found it arduous to work with MTA, as opposed to most other conventional dental materials [5]. Gel-form MTA is a novel material that is easier to handle and has enhanced washout resistance owing to its gel-based vehicles such as carboxymethyl chitosan or gelatin. A modified version of any conventional material is expected to give better material, with qualities that are either at par or superior. Conventional MTA possesses many important clinical applications such as direct and indirect pulp capping, repair of perforations, root-end filling, pulpotomy, repair of root resorption, apexification, coronal barrier, and as a filling material in root canals [6–8]. The pathogenesis of pulpal and periapical diseases is dominated by microorganisms and their by-products, therefore, the sealing ability and adaptability of material become crucial factors [6, 9]. Thus, this study was conducted to comparatively evaluate the sealing ability and adaptation of gel-form MTA to dentinal walls. The null hypothesis was set as there is no significant difference in the sealing ability and adaptation of gel-form MTA to dentinal walls to that of conventional MTA.

## Materials and methods

**Fig. 1:**
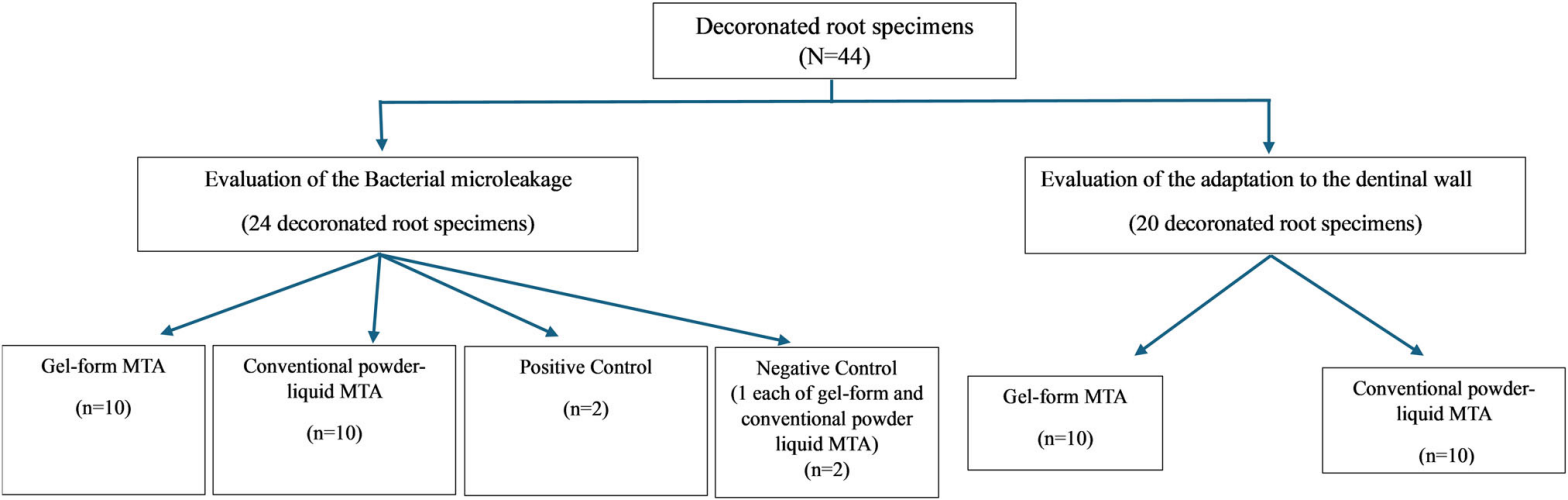
Flow chart illustrating the distribution of samples across the groups and tested parameters.

The in vitro experimental cross-sectional study was initiated after approval from Institutional Ethics Committee (Protocol reference number: 21066). The study was conducted on 44 permanent single-rooted teeth (incisors and canines having straight canals without cracks, caries, restoration, and resorption) extracted for therapeutic purposes, namely periodontal extractions in geriatric patients and extractions of supplementary teeth with full root length. Informed consent was obtained from the patients at the time of extraction for the storage and possible use of their extracted teeth for educational and research purposes. This was conducted as a pilot study with 10 samples per group and per each tested parameter. The sample distribution across the groups and tested parameters is illustrated in Fig. 1.

Tooth preparation: 44 selected teeth were immersed in 2.6% sodium hypochlorite (Septodont Healthcare India Pvt. Ltd., Maharastra, India) for 1 h to facilitate the removal of organic materials from the root surfaces. If any remnant tissue was noticed it was carefully removed using a curette. The teeth were then stored in physiologic saline (Realcade Lifescience Pvt. Ltd, Gujarat, India) to retain moisture. During the experiment, the teeth were decoronated using a bur mounted in a high-speed handpiece with water coolant to have a root length of 15 mm. An Access cavity was then prepared and the working length was assessed using a #15 K-file (Mani, Inc, Tochigi, Japan). The biomechanical preparation was carried out using a step-back technique to a #40 K-file (Mani, Inc, Tochigi, Japan) up to 3 mm shorter than the working length. The coronal portions were flared with the help of Gates-Glidden burs (Mani, Inc, Tochigi, Japan). To irrigate the canals during the canal preparation 2 mL of 5.25% sodium hypochlorite (Septodont Healthcare India Pvt. Ltd., Maharastra, India). The smear layer was removed with 5 mL of 17% EDTA (B.N. Laboratories, Mangalore, India) and finally, the canal was flushed with 5 mL of sodium hypochlorite. Once the instrumentation was completed, the canals were dried using sterile paper points.

Evaluation of the Bacterial microleakage: Out of 44, 24 specimens were used for evaluating the bacterial leakage. The gel-form MTA (Kids-e-dental LLP, Mumbai, India) and conventional powder-liquid MTA (Kids-e-dental LLP, Mumbai, India) were mixed as per the manufacturer’s recommendations. Of the 24 selected teeth, MTA mix was coronally introduced into the canals of 10 teeth in each group (total of 20 teeth) from an orthograde direction using MTA carrier up to 3 mm shorter than the estimated working length. The MTA was initially condensed using the thick end of moistened paper points and subsequently compacted with endodontic pluggers to create a 4 mm thick apical plug.

**Fig. 2:**
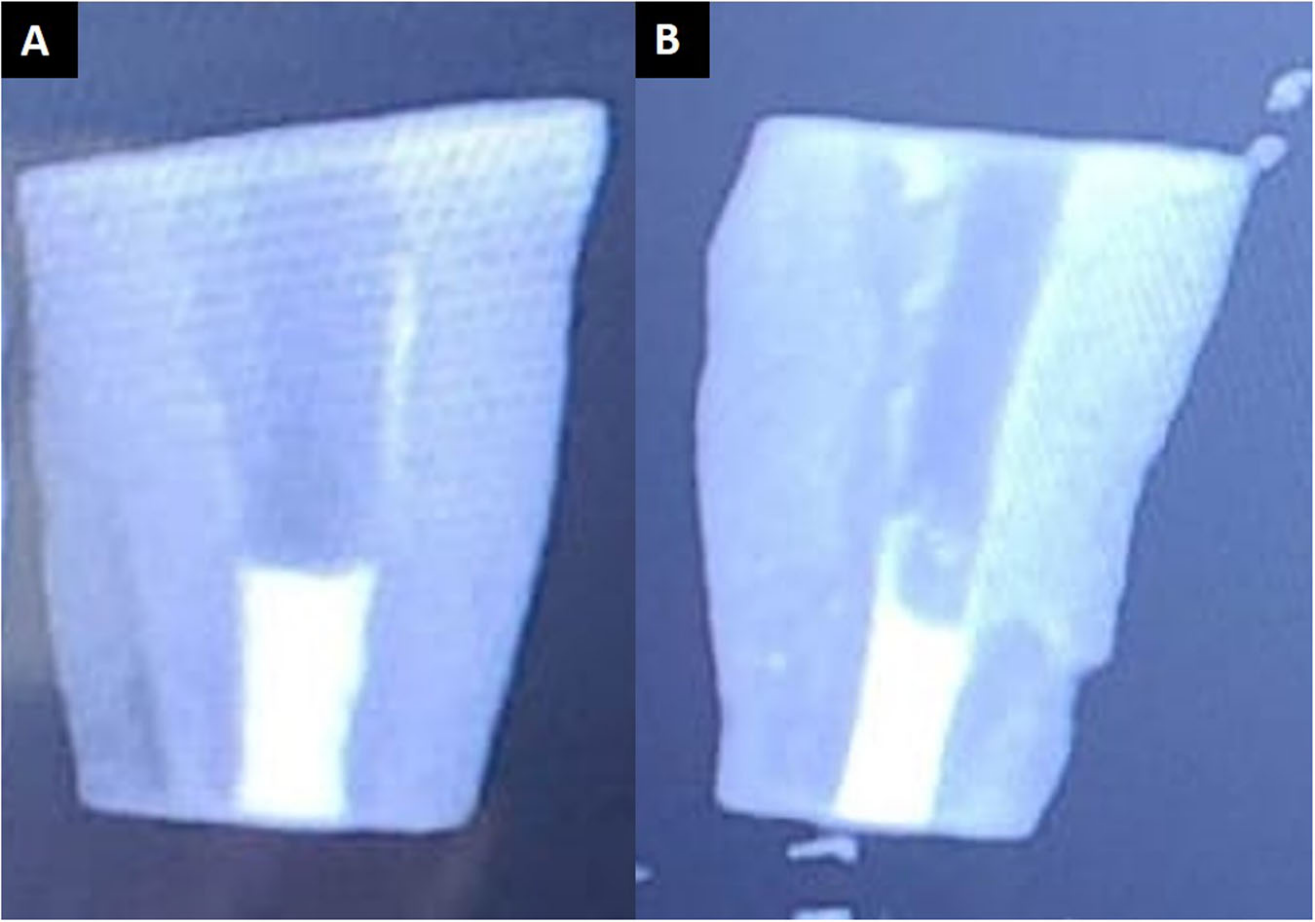
Radiographic images taken after filling the apical ends. **A** Conventional powder-liquid MTA. **B** Gel-form MTA.

To ensure void-free MTA placement and appropriate plug thickness digital radiographs were taken (Fig. 2). The remaining canal space was left unfilled, a moist cotton pellet was placed over the MTA, and the access was sealed using Cavit G (3 M™ ESPE, Germany). The samples were stored in a humidor at 37 °C and 95% humidity for 48 h. Later, the 4 mm root-end of the samples (10 samples from each group) was resected at 90 degrees to the long axis of the tooth, thus exposing the set MTA apical plug.

Out of the remaining 4 teeth, 2 teeth served as the negative control, and the other 2 were used as positive control. The negative control samples were obturated to the full length using MTA (one tooth for each group), while the positive control samples were instrumented but not obturated.

To prevent the possible bacterial microleakage through the accessory canals or other irregularities in the cementum, two coats of nail varnish were applied to the surfaces of all samples belonging to the experimental and positive control groups, excluding the resected apical portion and access cavity. In the negative control group, except for the access cavity, the entire root was painted with two coats of nail varnish [6].

**Fig. 3:**
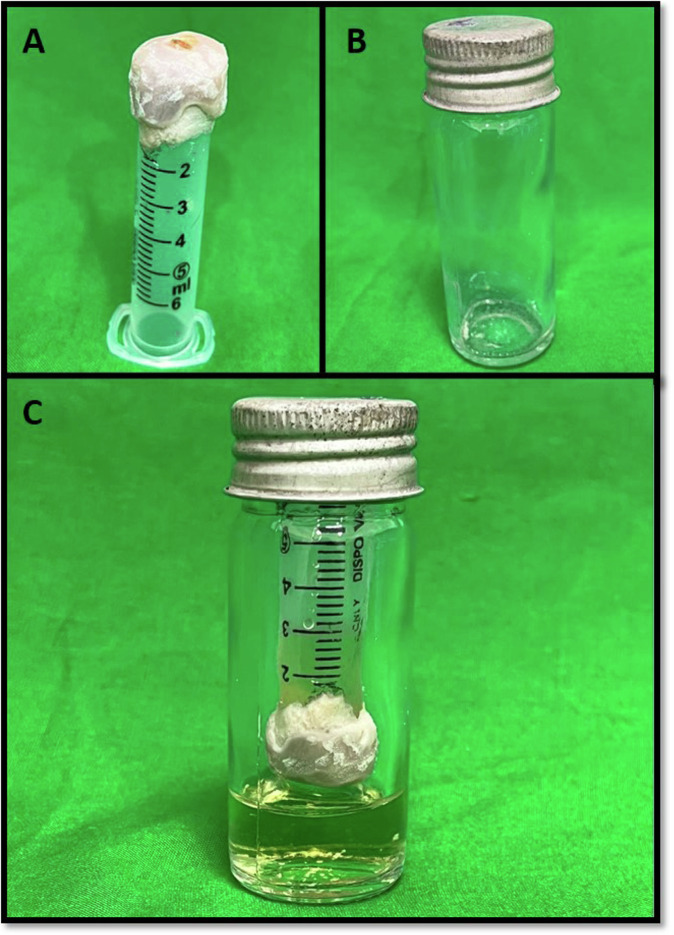
The bacterial microleakage apparatus. **A** The upper chamber of the apparatus was prepared using a plastic syringe and acrylic. The tooth is inserted such that the apical end is exposed. **B** The lower chamber of the apparatus- McCartney bottle. **C** The apparatus assembled together with sterile Muller Hinton broth in the lower chamber and upper chamber inoculated with *E. faecalis*.

The apparatus used to evaluate microbial leakage was a modified version of the apparatus previously described by Metgud et al. [10]. The apparatus was designed so that there would be a single pathway (root canal) between the upper chamber and the lower chamber. 5 ml disposable plastic syringes (Dispo Van, C S SurgiMed, Maharastra, India) were modified for use. The tips of the syringes were cut and fit with a rubber stopper. 1 mm diameter circular entry was made through the center of the rubber stopper using a high-speed air rotor handpiece into which the tooth was inserted in such a way that only the coronal end was inside the syringe barrel (upper chamber). Two layers of cyanoacrylate glue were applied to the interface between the tooth and the stopper to avoid direct bacterial penetration into the broth. One layer of cyanoacrylate glue followed by cold cure acrylic was applied at the interface between the rubber stopper and the plastic syringe to prevent contamination (Fig. 3A).

The components of the bacterial leakage model were autoclaved and then assembled under a laminar flow hood. The upper chamber was filled with Mueller Hinton Broth (Sisco Research Laboratories Pvt. Ltd., Maharashtra, India). A volume of 8–10 mL sterile Mueller-Hinton Broth was introduced into sterile McCartney bottles, as a lower chamber of the leakage apparatus (Fig. 3B). The previously assembled portions of the tooth (upper chamber) were then inserted in the McCartney bottles under an aseptic condition so that a minimum 2–3 mm of the apical part of each root is immersed in Mueller-Hinton Broth (Fig. 3C). To ensure sterilization, a sterility check was performed where the whole system was incubated at 37 °C for 3 days. Any test showing signs of turbidity in Muller- Hinton Broth was discarded. Two millimeters of Mueller-Hinton Broth was inoculated with 9 × 10^8^ CFU/mL (Mc Farland no.3) of *Enterococcus (E.) faecalis* (ATCC 29212) to form a bacterial suspension and that was added to the upper chamber under aseptic conditions, after which the McCartney bottles were tightly sealed. All 24 samples were placed in an incubator and observed every 2 days for turbidity of the broth in the lower chamber, which indicates bacterial growth. At the end of 30 days, the broth from the lower chambers of those samples exhibiting turbidity was cultured to observe the presence or absence of *E. faecalis*.

**Fig. 4:**
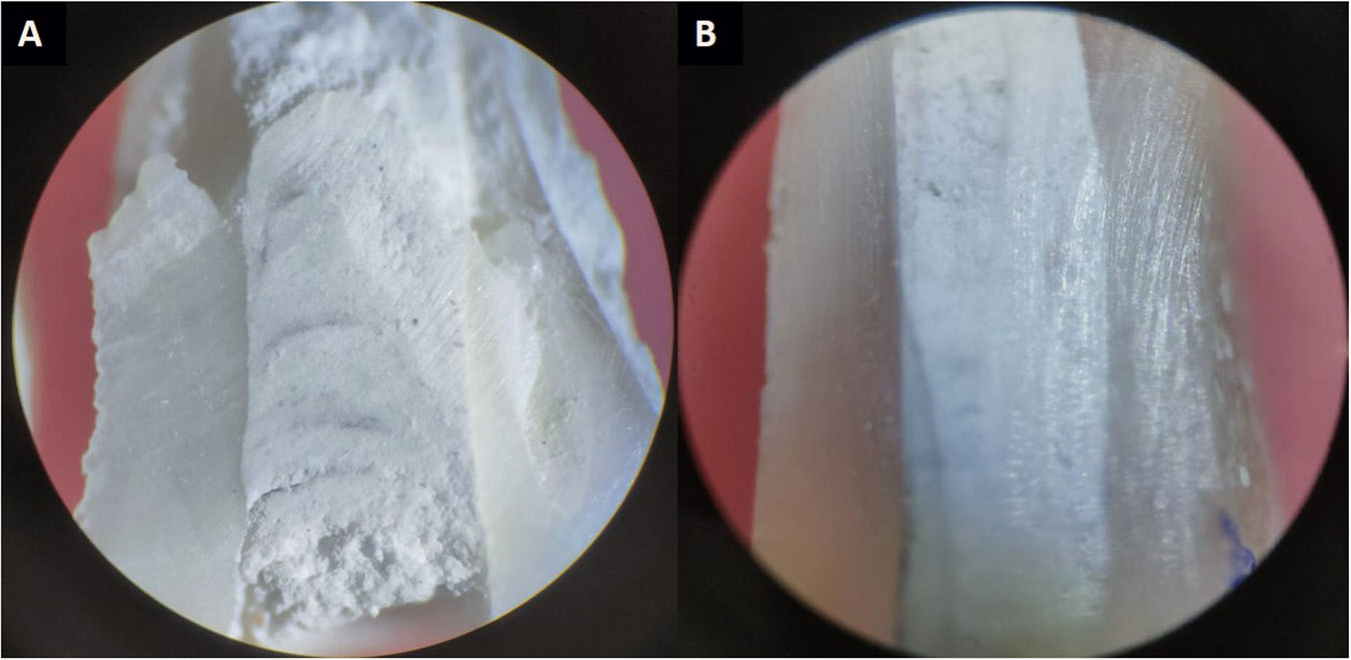
Longitudinally split sections of obturated teeth. **A** Conventional powder-liquid MTA. **B** Gel-form MTA.

Evaluation of the adaptation to the dentinal wall: The remaining 20 instrumented root specimens were obturated throughout their lengths using MTA (10 teeth with gel-form MTA and 10 teeth using conventional powder-liquid MTA). For obturation, the mixed MTA as per the manufacturer’s instructions was carried in a MTA carrier and placed in parts from the apical towards the coronal end of the root canal till CEJ while carrying out vertical condensation using a moist cotton pellet. Radiographs were taken to ensure the complete obturation of root canals with no voids. Once the material was completely set, root specimens were split longitudinally using a diamond disc in a high-speed handpiece and prepared for light microscopic (Olympus Magnus CH-20i, Navdurga scientific, New Delhi, Inia) evaluation (Fig. 4). Wax strips were placed on the microscopic slides to provide stability to the longitudinally split teeth. The gaps between the MTA and canal walls were detected and measured in coronal, middle, and apical thirds [9]. The gap sizes were recorded in square millimeters using Image J software.

### Statistical analysis

To assess the differences in gaps at various levels of the root, an independent t-test was performed to compare gel-form MTA with conventional powder-liquid MTA. Intra-group comparison of the gaps identified at different levels of the root (apical, middle, and coronal) was done using the Friedmans test. To describe data related to bacterial leakage, frequency, and percentage were used with a chi-square test. Statistical analysis was conducted using SPSS 20.0, and a *p* < 0.05 was considered statistically significant.

## Results

While evaluating the adaptation properties, we identified gaps in 9 out of 10 samples of both gel-form MTA and conventional powder-liquid MTA at the coronal and middle thirds. At the apical one-third, 8 samples of gel-form MTA and 7 samples of conventional MTA demonstrated the presence of gaps (Table 1). On comparing the adaptation property of the gel-form MTA with that of the conventional powder-liquid MTA, at coronal and apical thirds, no statistically significant difference was found (*p* = 0.071 and *p* = 0.638 respectively). However, while comparing the same in the middle one-third of the root a statistically significant difference was found (*p* = 0.013) between the two groups, with lesser gaps being identified in the MTA gel group.

**Table 1:** Intergroup comparison of the gaps identified at various levels of the root between gel-form MTA and conventional powder-liquid MTA using an Independent t-test.

Root level	Group	Number of samples with a gap	Gap size Mean ± SD	t-value	*P*-value
Coronal	Gel-form MTA (*n* = 10)	9	0.006 ± 0.01	−2.073	0.071
	Conventional powder-liquid MTA (*n* = 10)	9	0.054 ± 0.069		
Middle	Gel-form MTA (*n* = 10)	9	0.004 ± 0.004	−3.138	0.013^*^
	Conventional powder-liquid MTA (*n* = 10)	9	0.032 ± 0.027		
Apical	Gel-form MTA (*n* = 10)	8	0.006 ± 0.008	−0.481	0.638
	Conventional powder-liquid MTA (*n* = 10)	7	0.008 ± 0.008		

^*^P < 0.05 is statistically significant.

When the material adaptation was compared among coronal, middle, and apical root levels within the group (intra-group comparison) using Friedmans test (Table 2), no statistically significant difference was found across the different root levels for both gel-form MTA and conventional powder-liquid MTA (*p* values 0.882 and 0.651 respectively).

**Table 2:** Intra-group comparison done comparing gaps identified at different levels of the root using Friedmans test.

	Gaps identified at	*n*	Mean (SD)	Median (25th, 75th percentile)	Range	Mean Rank	Chi-square value	*P* value
Gel-form MTA	Coronal	10	0.006 (0.01)	0.003 (0.002, 0.004)	0.001–0.032	2.13	0.25	0.882
	Middle	10	0.004 (0.004)	0.002 (0.001, 0.006)	0–0.012	1.88		
	Apical	10	0.006 (0.008)	0.003 (0.002, 0.008)	0.0003–0.024	2		
Conventional powder-liquid MTA	Coronal	10	0.054 (0.069)	0.006 (0.001, 0.102)	0–0.172	2.14	0.857	0.651
	Middle	10	0.032 (0.027)	0.014 (0.013, 0.048)	0.01–0.077	2.14		
	Apical	10	0.008 (0.008)	0.007 (0.001, 0.015)	0.001–0.024	1.71		

When bacterial leakage was compared between the groups, turbidity was present in one sample belonging to the conventional powder-liquid MTA group and the noted difference was statistically significant (Chi-square value = 15.771 & *p* = 0.001) (Table 3). A negative result was seen when the turbid sample belonging to the conventional powder-liquid MTA group underwent a catalase test. Regarding colony counts for Enterococcus faecalis, gel-form MTA and the negative control had no bacterial contamination (0%), while the powder-liquid MTA showed 10% contamination, and the positive control had 50% contamination with *E. faecalis*. Additionally, positive control had 50% contamination with other microbial species. The chi-square value of 18.171 and a *p* value of 0.006 further support the significant differences between the groups in terms of bacterial growth and contamination.

**Table 3:** Percentage distribution table representing the bacterial leakage seen in the form of turbidity, catalase test results and colony count results as applicable.

Parameter evaluated		Gel-form MTA *n* (%)	Conventional Powder-liquid MTA *n* (%)	Positive Control *n* (%)	Negative Control *n* (%)	Chi-Square values	*P* value
Turbidity	Absent	10 (100)	9 (90)	0 (0)	2 (100)	15.771	0.001
	Present	0 (0)	1 (10)	2 (100)	0 (0)		
Catalase test	Negative	0 (0)	1 (10)	2 (100)	0 (0)	15.771	0.001
	No colonies	10 (100)	9 (90)	0 (0)	2 (100)		
Colony Count	*E. faecalis* present- 10^3^ colony forming units/milliliter	0 (0)	1 (10)	1 (50)	0 (0)	18.171	0.006
	*E. faecalis* present, along with other microbial contamination	0 (0)	0 (0)	1 (50)	0 (0)		
	Not applicable	10 (100)	9 (90)	0 (0)	2 (100)		

## Discussion

MTA is a calcium silicate-based cement, that potentially exhibits improved washout resistance when carboxymethyl chitosan or gelatin is incorporated thereby enhancing its bioactivity and sealing properties [11, 12]. This innovation comes from the construction industry, which also uses Portland cement as a binder similar to MTA [13]. A water-soluble polymer admixture is added to enhance the rheological properties and resistance to the washout of the Portland cement binder during the construction of underwater structures [14]. The e-MTA gel is a novel MTA formulated using the same technology.

MTA in dentistry has got numerous applications [15]. In the present study, we used gel-form MTA for creating an apical plug as in apexification and evaluated the sealing ability in comparison with the conventional powder-liquid MTA. The results of the present study showed less bacterial leakage in the gel-form MTA group. This may be because of the smoother consistency of the gel-based MTA attributed to the ultrafine-grained powder as claimed by the manufacturers. Moreover, it’s said that the gel form has superior physical and chemical properties to the distilled water type [16].

The most commonly used methods of evaluating the sealing ability of root-end filling materials are autoradiography and dye penetration techniques. The former is a subjective and quantitative method, but the results obtained are dependent on various factors like the type of isotope, the distance between the radiation source and emulsion, and the length of exposure of the film [17]. In addition, the validity of this method is questioned by Matloff et al. [18], as radioisotope tracers being smaller than bacteria are likely to have dissimilar leakage patterns. Similarly, the popular and user-friendly dye penetration method also has the following shortcomings: (a) smaller molecular size of the dye particles penetrate deeper than the bacteria; (b) total leakage is not measured as the degree of leakage is measured only in one plane; and (c) lack of reproducibility [19, 20]. Considering the drawbacks of the above-said methods, we used bacterial leakage as the method to test the sealing ability as suggested by Torabinejad et al. [21].

The present study also evaluated the adaptation of e-MTA gel to the root canal walls by identifying and measuring the gaps present at the coronal, middle, and apical thirds of the root. The results showed lesser gaps present in the middle third of the samples belonging to the gel-form MTA group. This finding supports the results of the bacterial leakage test. Better adaptation of the gel-form MTA might have resulted in a better seal in the gel-MTA group. Supporting the above-mentioned findings, a study conducted by Brito-Júnior et al. [22] reported significantly lower leakage when gray MTA-Angelus was mixed with propylene glycol in comparison to when mixed with distilled water for repairing the furcal perforations. The study used a single aerobic bacterial leakage test similar to the present study.

However, there is limited literature comparing the bacterial leakage and adaptation of gel-form MTA to the conventional powder-liquid MTA. The present study was conducted under in-vitro conditions using a single aerobic leakage model which doesn’t completely simulate the intra-oral environment and is the limitation of the study. Thus, future studies are recommended to overcome the said limitation and also to synthesize substantial evidence in association with gel-MTA usage.

## Conclusion

Considering the limitations, the present study concludes that gel-form MTA shows a better adaptation to the dentinal walls at the middle third of the root and exhibits better sealing ability against bacterial leakage when tested for *E. faecalis*. The adaptation of gel-form MTA at coronal and apical third of the root was comparable to the conventional powder-liquid MTA.

## Data Availability

The original data is available with the corresponding author and can be shared on request.
